# Instrument-Free Protein Microarray Fabrication for Accurate Affinity Measurements

**DOI:** 10.3390/bios10110158

**Published:** 2020-10-29

**Authors:** Iris Celebi, Matthew T. Geib, Elisa Chiodi, Nese Lortlar Ünlü, Fulya Ekiz Kanik, Selim Ünlü

**Affiliations:** 1 Department of Electrical and Computer Engineering, Boston University, 8st Mary’s Street, Boston, MA 02215, USA; geib@bu.edu (M.T.G.); elich@bu.edu (E.C.); fulyaek@bu.edu (F.E.K.); 2 Department of Biomedical Engineering, Boston University, 8st Mary’s Street, Boston, MA 02215, USA; nunlu@bu.edu

**Keywords:** label-free biosensor, binding kinetics, affinity measurements, protein microarray

## Abstract

Protein microarrays have gained popularity as an attractive tool for various fields, including drug and biomarker development, and diagnostics. Thus, multiplexed binding affinity measurements in microarray format has become crucial. The preparation of microarray-based protein assays relies on precise dispensing of probe solutions to achieve efficient immobilization onto an active surface. The prohibitively high cost of equipment and the need for trained personnel to operate high complexity robotic spotters for microarray fabrication are significant detriments for researchers, especially for small laboratories with limited resources. Here, we present a low-cost, instrument-free dispensing technique by which users who are familiar with micropipetting can manually create multiplexed protein assays that show improved capture efficiency and noise level in comparison to that of the robotically spotted assays. In this study, we compare the efficiency of manually and robotically dispensed α-lactalbumin probe spots by analyzing the binding kinetics obtained from the interaction with anti-α-lactalbumin antibodies, using the interferometric reflectance imaging sensor platform. We show that the protein arrays prepared by micropipette manual spotting meet and exceed the performance of those prepared by state-of-the-art robotic spotters. These instrument-free protein assays have a higher binding signal (~4-fold improvement) and a ~3-fold better signal-to-noise ratio (SNR) in binding curves, when compared to the data acquired by averaging 75 robotic spots corresponding to the same effective sensor surface area. We demonstrate the potential of determining antigen-antibody binding coefficients in a 24-multiplexed chip format with less than 5% measurement error.

## 1. Introduction

Understanding the true nature of the interaction between ligands and analytes requires accurate analysis of the underlying binding kinetics, necessitating label-free detection (LFD) techniques to study biomolecular interactions [1]. In addition to measuring the binding affinity, it is often desirable to quantify the specificity of the interaction and cross-reactivity of the analyte to similar ligands, especially for proteins. Thus, multiplexed LFD methods have emerged as essential laboratory techniques. Solid-phase assays for LFD, especially in optical imaging modality, can interrogate arrayed sensor surfaces, and thus, benefit from advances in protein microarray fabrication. Both quantitative and functional proteomics have been exploiting the advantages of microarrays for a well-controlled study of proteins [2,3]. Protein microarrays offer multiplexing and low sample volume consumption, drastically reducing the time and resources required for sample analysis with respect to other methods [4].

To fully exploit the advantages of dense protein microarrays, binding kinetics analysis platforms, such as Surface Plasmon Resonance (SPR), utilize external custom microarray spotting services or in-house robotic microspotters (SPRi-Continuous Flow Microspotter, SPRi-Arrayer, Arrayjet, M2 instrumentSIX). These arraying instruments offer precise sample dispensing of extremely low solution volumes, and, can therefore, be utilized to create arrays with hundreds to thousands of probe spots; however, they are often bulky, complex, and expensive. Therefore, the multiplexed affinity measurements for proteins remain elusive for laboratories with limited infrastructure and resources. For those applications, for which this level of precision and multiplexing may be unnecessary, a manual (instrument-free) arraying capability along with a compatible LFD technique is highly desirable.

The Interferometric Reflectance Imaging Sensor (IRIS) platform is a thin-film interference-based technology for dynamic mass accumulation measurements for label-free binding kinetics analyses. The IRIS system is a simple common path interferometer utilizing multi-color LED illumination and a CMOS camera for imaging a large (>30 mm^2^) sensor surface. The IRIS sensors are Si chips with a thin layer of thermally-grown oxide on top, allowing for scalable fabrication. The instrumentation and chip consumables are low-cost and accessible for smaller research labs without significant capital investment. However, there is a bottleneck regarding surface immobilization of molecular probes. Traditionally, robotic microarray spotters have been used to create high-density (hundreds of spots) multiplexed sensor surfaces with homogenous surface morphology. For high-sensitivity measurements, especially for low molecular weight (MW) target molecules, spatial averaging was employed on replicate spots for maintaining statistical significance and achieving a low noise level. While robotic printing produces molecular spots with excellent surface morphology, it introduces a high-cost infrastructure element into the affinity measurement workflow, and a low-cost alternative would be highly desirable.

Here, we present a method by which a researcher familiar with dispensing samples through a micropipette can rapidly create a multiplexed protein array with as many as 24 different probes on an IRIS chip that maintains excellent sensitivity and capture efficiency. An SNR analysis of label-free binding kinetics assays is performed, and the results obtained by instrument-free arraying are compared favorably to those obtained for a microarray fabricated through an automated robotic spotter. We finally demonstrate that this instrument-free microarray fabrication method significantly reduces the cost and resources necessary for producing a multiplexed affinity measurement workflow, combined with high-sensitivity and small molecule measurement capability offered by IRIS technology. Thus, accurate multiplexed affinity measurements, once limited to high-resource laboratories, will be accessible for individual researchers and small research laboratories.

## 2. Materials and Methods

### 2.1. The IRIS Platform

**Figure 1:**
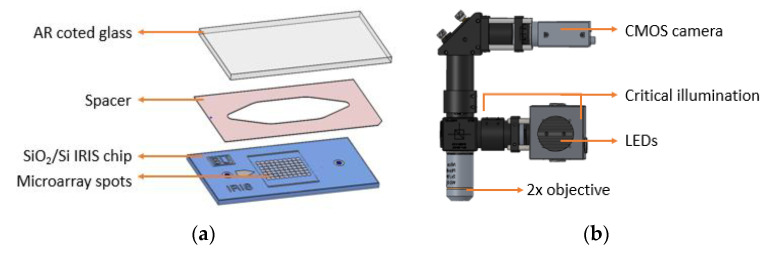
(**a**) The Reflectance Imaging Sensor (IRIS) fluidic cartridge; (**b**) the IRIS optical setup.

**Figure 2:**
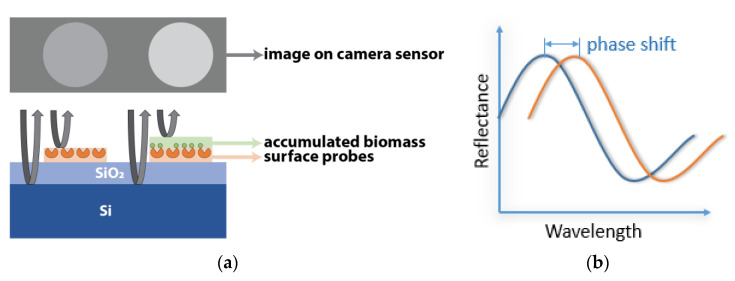
IRIS working principle; (**a**) demonstration of biomass accumulation on the sensor surface and (**b**) the resulting change in reflectance of the film.

The principles that drive the IRIS system and the advantages it offers have been described extensively [4,5,6]. Briefly, the IRIS system (Figure 1) works on the principle of thin-film (110 nm SiO_2_/Si) interference and label-free detection of biomass accumulation with a CMOS camera. In this common path interferometer configuration, biomass accumulation on the sensor surface causes an effective increase of the top transparent layer (oxide). The change in optical path difference (OPD) results in a spectral shift in reflectance, affecting the recorded intensity of the image (Figure 2). To convert intensities to mass accumulation, four images are taken before the experiment at 457 nm, 518 nm, 595 nm, and 632 nm with narrowband LEDs, and a lookup table is created for each sensor surface [7]. During experimentation, the sensor is illuminated with a single 457 nm LED.

The full assembly of the microfluidic cartridge consists of placing a 50 µm imaging spacer (Grace Bio-Labs, Bend, OR) between the SiO_2_/Si IRIS chip and an anti-reflection (AR)-coated glass slide with inlet/outlet through holes, creating a sealed fluidic chamber.

### 2.2. Image Acquisition and Analysis

The real-time videos were captured with the CMOS camera FLIR BlackFly BFS-U3-70S7M-C. To maximize the sensitivity of the IRIS platform, the dominant noise factors should be minimized. In a bright field imaging setup, such as IRIS, the predominant noise contributor is shot noise. Shot noise or Poisson noise originates from the discrete nature of electrons, and can be modeled with a Poisson distribution. Since the variance of a Poisson distribution is equal to the number of events, in this case, electrons, in a shot noise limited system, the SNR increases with the square root of the number of generated electrons. The number of electrons accumulated on a single pixel is physically limited by its full well capacity, and therefore, SNR improvement by merely maximizing the accumulation is insufficient. In the earlier work, we have demonstrated SNR improvement, due to averaging nearly identical robotically spotted capture probes and temporal averaging [8]. In this study, we start with the same premise, and we demonstrate that noise is further decreased with increase spot area. The regions of interests include the spatially averaged spot area and its neighboring spatially averaged background area. Analyses of binding characteristics and SNR levels are performed in MATLAB.

### 2.3. Sensor Chip Surface Functionalization

Sensor functionalization acts to capture and covalently bind probes to the sensor surface without hindering their activity. We prepared our chips with a DMA-NAS-MAPS-based polymeric coating (commercially known as MCP-2, Lucidant Polymers LLC) [9]. Before coating, the chips were plasma cleaned with strong air plasma for 10 min to activate the silicon dioxide surface. The cleaned chips were submerged in a 1% *w*/*v* MCP-2 polymer in a 10% saturated ammonium sulfate solution. These chips were then incubated on a shaker plate for 30 min, washed by swirling in 1 L of MilliQ water, dried under a nitrogen stream, and placed into a vacuum drying oven at 80 °C for 15 min. The chips are then placed into a vacuum desiccator for at least 1 h prior to use. 

### 2.4. Probe Preparation and Spotting

**Figure 3:**
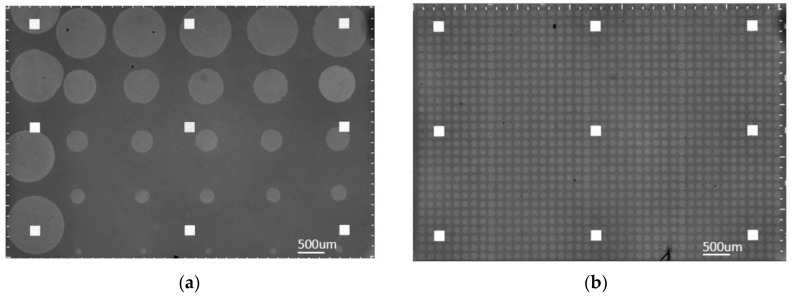
Spotted IRIS chips; (**a**) from left to right, one column of micropipette spots followed by five columns of robotically deposited spots in decreasing number of droplets per spot; (**b**) identical robotically dispensed single droplet spots (30 × 40 array).

Before spotting, all coated chips were placed in a humidity chamber set to 57% humidity for 10 min. Both BSA and α-lactalbumin proteins were diluted to a concentration of 1 mg/mL with 1X-PBS (22 µm-filtered before use). As seen in Figure 3a, a partial array of α-lactalbumin was spotted manually on one side of the sensor chip using a micropipette set to 0.25 uL (spot size ~1000 µm), while the array of spots with sizes ranging from 130 µm to 1000 µm was created with robotic spotting, by varying the number of 120 pL droplets dispensed by M2 iTWO™-300P Microarray Spotter at 57% humidity, on both MCP-2 coated chips. For more accurately spaced arrays, a rudimentary X-Y stage can be used for manual spotting. The spotted chips were then incubated at 67% humidity overnight. Upon retrieving these chips from the humidity chamber, MCP-2 coated chips were blocked in a solution of 150 mM ethanolamine and 100 mM Tris at pH 9 for one hour. All experiments were performed with both freshly prepared and month-old batches; we did not observe any degradation or difference in capture efficiency.

As seen in Figure 3b, a robotic spotting scheme was implemented for direct comparison. This scheme consisted of α-lactalbumin at 1 mg/mL and 0.5 mg/mL concentration, as well as negative control columns consisting of BSA at 1 mg/mL concentration. Although the lower concentration spots were used for binding consistency validation, this study focuses on the higher concentration spots. These chips were produced using the M2 iTWO™—300P robotic spotter, at 57% humidity, in the same run as the chips mentioned above to maintain consistency.

### 2.5. Chemical and Biological Materials

All reagents and buffers were purchased from Sigma Aldrich (St. Louis, MO, USA). Bovine serum albumin (BSA) and α-lactalbumin were purchased from Sigma Aldrich (St. Louis, MO, USA). The anti-α-lactalbumin antibody was purchased from Abcam (Cambridge, UK). All protein and antibody samples were diluted by 1X-PBS. MCP-2 was purchased from Lucidant LLC. P-doped silicon wafers with thermally grown silicon dioxide were purchased from Silicon Valley Microelectronics (Santa Clara, CA, USA).

## 3. Results

### 3.1. SNR Analysis of α-Lactalbumin—Anti-α-Lactalbumin Binding Curve: Dense Array, Single Droplet Spots

**Figure 4:**
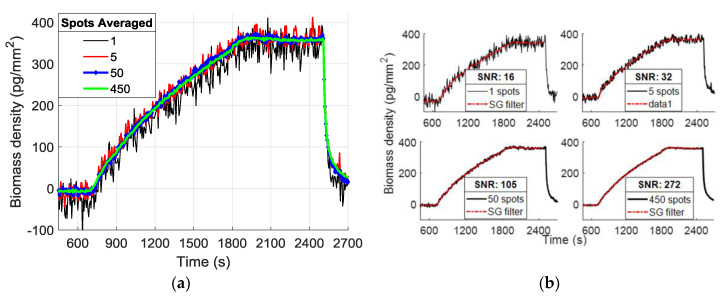
(**a**) Example binding curves from MCP-2 functionalized chip with single drop 1 mg/mL α-lactalbumin spots showing the effect of spatial averaging. (**b**) Binding curves with Savitzky–Golay filtering and their calculated SNR level.

To calculate the SNR of the binding curves, a definition of the system’s signal is needed, followed by the extraction of the noise level. Here we define binding as the mass accumulation on the spot area, calculated from the thickness difference between the spot and its corresponding background, hence, the difference in intensity readings between the spot and the background. The binding signal is defined as the differential intensity, converted to an accumulated number of electrons on the camera sensor. We know that in a shot noise limited system, the SNR level will be proportional to the square root of the total number of electrons (N) accumulated on the CMOS camera sensor, since the signal level is proportional to N and the dominating noise (shot noise) will be inversely proportional to $\sqrt{N}$. The primary goal in binding experiments is extracting the binding coefficients of antigen-antibody complexes. Hence, our definition of the signal is slightly different from the classic idea of absolute intensity signal measured on the CMOS sensor; we aim to demonstrate the sufficient SNR level in an experiment for accurate estimation of binding characteristics. For this reason, after recording the signal level, we apply a Savitzky—Golay filter to binding-debinding events to estimate what we define as the “noiseless” curve for each averaging step. Then, we extract the noise by calculating the standard deviation of the difference between raw data and the estimated noiseless curve. Lastly, the SNR is calculated as the level of the binding signal divided by the level of extracted noise. The binding experiment for a dense array of single 120 pL spots of probe solution, as seen in Figure 3b, was run for the fabricated chips. Figure 4a illustrates the binding curves for a single spot, and also a random selection of 5, 50, and 500 spots curves averaged together; Figure 4b shows the filtered curves and the calculated SNR for each curve.

**Figure 5:**
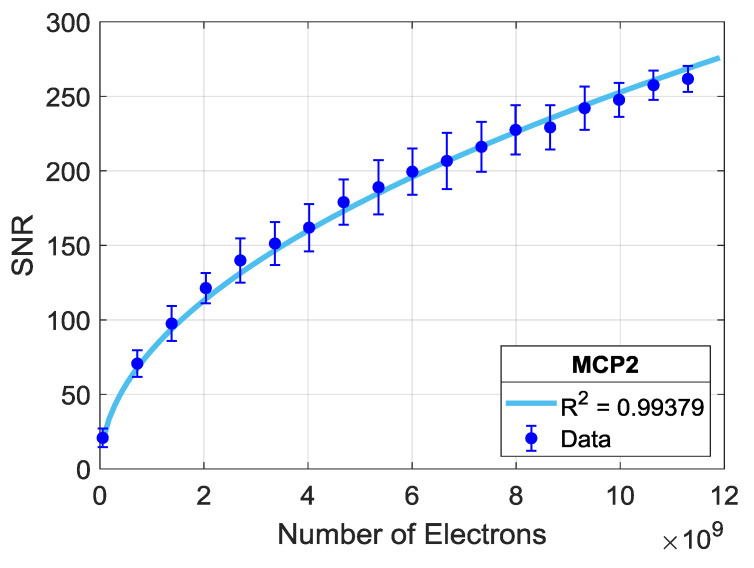
SNR dependence on a number of total electrons, captured from averaging a growing number of identical spots on MCP-2 functionalized chips; 1 mg/mL ligand (α-lactalbumin) concentration. The data points are fitted with a square root law, and the R^2^ value is shown.

The SNR analyses for binding curves extracted from spatially averaged spots on different chips are shown in Figure 5. Here, for each number of averaged spots (n) (on each step increasing n), we apply the explained SNR calculation on multiple (>100) random selections of n spots. The median of the calculated SNR values for each n is fitted to a square root and power curve, and the median absolute deviation (MAD = median (|x − median(x)|)) for each n is illustrated with error bars. Medians and MADs are plotted with respect to the corresponding total number of electrons averaged. The applied curve fitting reveals that SNR increases in a square root dependence on the accumulated number of electrons with an R^2^ of 0.99.

### 3.2. SNR Analysis Comparison of Spots with Varying Areas and Micropipette Spots 

**Figure 6:**
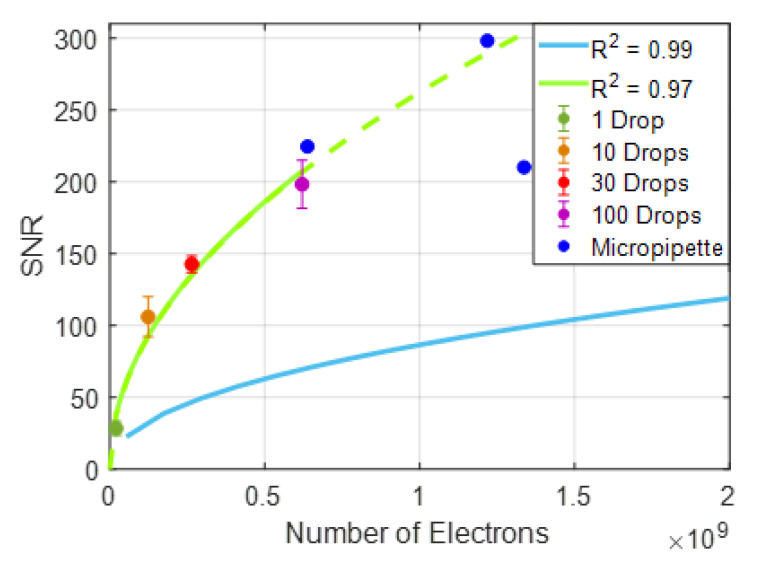
Comparison of the SNR levels obtained from MCP-2 functionalized chips. Averaged identical robotic spots (blue—previously shown), robotic spots of increasing size, and micropipette spots, with respect to the total number of electrons collected from the spots. The SNR behavior of larger robotic spots is also fitted to a square law curve (green).

In the previous section, an analysis of accumulated electron dependence of SNR was demonstrated for α-lactalbumin—anti-α-lactalbumin binding experiments with the dense array of robotic spots. Single droplet (120 pL) spots were averaged to increase the area of spatial averaging, effectively increasing the number of electrons. A similar SNR analysis was performed on chips robotically spotted with a varying number of droplets per spot, hence, varying areas; and manually spotted with large surface areas by micropipetting (Figure 5). In Figure 6, the calculated SNRs of different sized spots, the variations within the same spot size, and previously obtained SNR behavior of dense array single drop spots are plotted together. The SNR levels of spots with larger areas are observed to be higher than that of the multiple single droplets averaging with the same total electron yield. The SNR dependence on electrons this time was shown for the robotically-created bigger spots, with a square root curve fitting having an R^2^ of 0.97.

**Figure 7:**
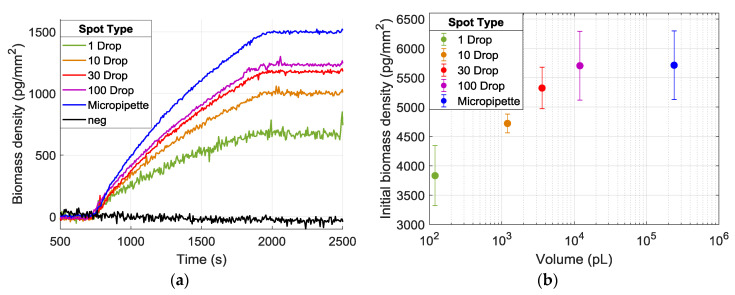
**(a)** Binding curves from spots created with dispensing 1–100 droplets and micropipette spots. Each curve is obtained from a single spot within its duplicates, having the median accumulation level. (**b**) The calculated thickness of spots and the corresponding volumes dispensed.

The improvement in the SNR levels results from the higher signal levels observed in spots having a larger area. Figure 7a demonstrates the increasing signal level with increasing spot size. Clearly, the amount of mass accumulation on the surface depends on the probe density and efficiency of each spot, and increasing the dispensed volume on the surface is expected to increase the number of probes per unit area, until saturation of the surface [10]. Figure 7b shows the initial thicknesses of spots with varying sizes; hence, the immobilized probe densities, and the mass accumulation obtained from the experiment.

Although measuring immobilized probe density demonstrates the capture capability of the microarray spot, it is not adequate to assess the performance. This is precisely the reason for our comparison using kinetic binding curves and error in fitting binding coefficients.

### 3.3. Variance and Percent Error in Extracted Binding Coefficients with Respect to SNR Levels 

**Figure 8:**
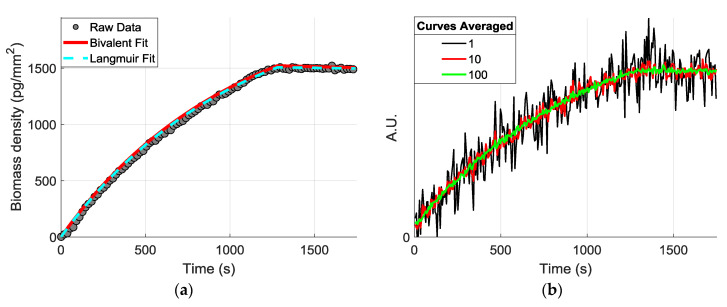
(**a**) Example raw data from obtained is shown with a Bivalent and Langmuir fit, with R^2^ values of 0.998 and 0.993, respectively. (**b**) The theoretical curves were created from the extracted coefficients with an additive Poisson noise.

As we mentioned before, the primary goal of many binding experiments is the correct estimation of binding coefficients between the analyte and the ligand. A bivalent antibody may bind to two separate antigens, and the binding of antigen to one binding site may directly impact the efficiency of binding to the other binding site on the same antibody. This Bivalent model is described as follows [11]:

(1) $$\left[L\right]+2\left[R\right]\underset{k_{\mathrm{off}1}}{\overset{k_{\mathrm{on}1}}{↔}}\left[LR\right]+\left[R\right]\underset{k_{\mathrm{off}2}}{\overset{k_{\mathrm{on}2}}{↔}}\left[LRR\right]$$

where k_on1_ and k_off1_ represent the first association and dissociation rates, and similarly, k_on2_ and k_off2_ are the secondary association and dissociation rates. To understand the binding kinetics of these interactions, the more computationally efficient Langmuir model can also be used [12]:

(2) $$\left[L\right]+\left[R\right]\underset{k_{\mathrm{off}}}{\overset{k_{\mathrm{on}}}{↔}}\left[LR\right]$$

where k_on_ and k_off_ represent the association and dissociation rates for the single-valent interaction of two molecules. This single-valent model offers a simpler analytic solution and is frequently used to model the antigen-antibody binding characteristics, since the secondary association and primary dissociation rates (k_on2_ and k_off1_) can usually be neglected—for high-affinity reactions—in favor of the primary association and secondary dissociation rates (k_on1_ and k_off2_). To demonstrate the effect of SNR improvements on these estimations, we first applied the Langmuir fitting to extract the binding coefficients and simulate a theoretical curve, and we simulated different “noisy” curves by adding a random noise component having a Poisson probability distribution (Figure 8).

**Figure 9:**
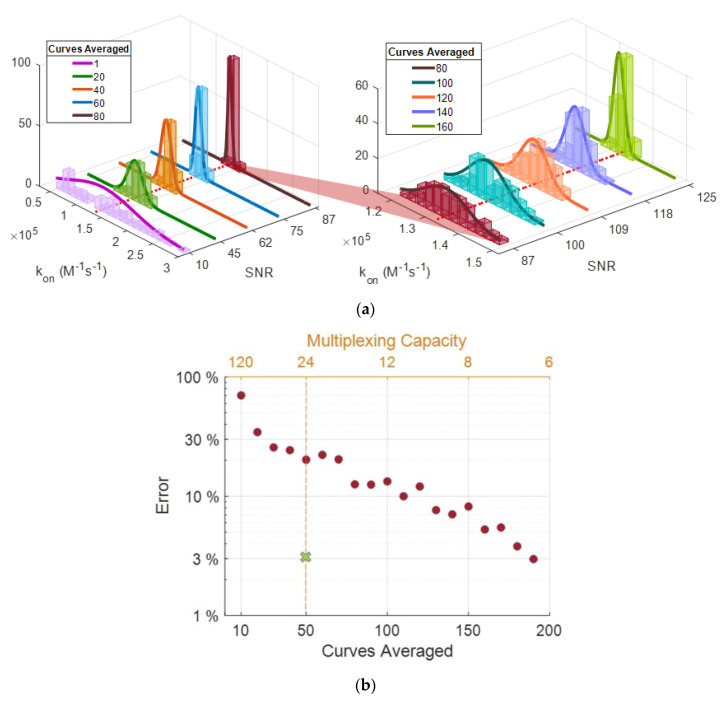
(**a**) Histograms for the extracted k_on_ values with a corresponding number of curves averaged and the yielding SNR values (SNR (**a**) 10–87, (**b**) 87–125). The red-dotted line represents the actual calculated k_on_ value (1.33 × 10^5^). (**b**) The highest percent error was observed from each group of kon estimations from averaging steps. The marked spot on the plot (green) represents the projected percent error from micropipette spots on the 24-multiplexing line.

We then averaged a growing number of curves (n), each step having a hundred random selections of n different curves to mimic the improvement of SNR by spatial averaging. For every averaged curve, the Langmuir fitting was performed, and the k_on_ and k_off_ rates were recorded for each random selection. Figure 9 shows the histograms of the different k_on_ coefficients extracted from each random selection of n averaged curves.

The histogram in Figure 9a displays that increasing SNR enables greater precision in predicting k_on_ values for α-lactalbumin and anti-α-lactalbumin association coefficient. The deviation of estimated binding coefficients from expected values drastically decreases with increased SNR of the curves; for k_on_, k_off,_ and k_d_, a maximum percent error of 129, 553, and 1524 were observed for initial curves (SNR~10), and 2.3, 54, and 53 were observed after averaging 150 curves (SNR~123), respectively. Figure 9b demonstrates the decrease in percent error with averaging (with the increase of SNR); we also show the estimated percent error (~3%) from micropipette spots as performance comparison. With a rudimentary XY stage, an array of 6 × 4 spots (~1000 μm diameter) can be easily placed on the sensor surface, yielding a multiplexing capacity of 24.

## 4. Discussion

In this study, we have compared the efficiency of robotically spotted microarrays and instrument-free created spots for the α-lactalbumin protein and anti-α-lactalbumin antibody binding experiment. Given that the IRIS platform is a shot noise limited system, we expect to have an increased SNR, therefore, increasing accuracy and sensitivity with spatial averaging. The performed SNR analyses revealed that averaging performed on the single drop assays follows the $\sqrt{N}$ rule, showing the improvement of increasing the effective area/total electron accumulation. Four hundred fifty replicate spots averaged showed an SNR of 260. Based on this result, the spots with larger surface areas (hence, the increased number of electrons) were analyzed and compared with the same method. We observed an improved signal level for the larger spots (robotic and instrument-free), with respect to averaging duplicate single drop spots with the same total electron yield for both surface coatings. Micropipette spots having the same area/electron yield of averaging 50–75 single drop duplicate drops (SNR level ~ 70–110) showed SNR levels of 210–298. As expected, the increased volume dispensed (proportional to radius cubed) on the surface resulted in an increase in probe density within the spot area (proportional to radius squared). Probe density, due to the saturation of the spot surface, remained the same after 10 nL. We then demonstrated the effect of noise level on the accurate extraction of binding coefficients by first fitting a binding curve obtained from the kinetic measurements (k_on_ = 1.33 ×10^5^ k_off_ = 1.53 × 10^−5^), and then simulating noisy curves with the same parameters and additive Poisson noise. The variance of the estimated parameters decreased with an increasing number of curves averaged (therefore increased SNR) and the percent error of the values decreased by 55, 10, and 28-fold for k_on_, k_off_, and K_d_, respectively.

In conclusion, we demonstrated the excellent performance of the instrument-free assay fabrication method in comparison to robotic spotting. Micropipette spots achieved higher signal levels (2.8–4.3 fold) and higher SNR levels (~3 fold) than the averaging of robotic spots with the same area yield (~75 spots). Coupled with the IRIS platform, this method enables accessibility of high-sensitivity affinity measurements in a multiplexed manner for small research laboratories.
